# Multilevel support for continued physical activity in university volleyball courses: a qualitative study

**DOI:** 10.3389/fpubh.2026.1960528

**Published:** 2026-09-25

**Authors:** Huiteng Xu, Qianqian Fan, Seo Yeonhee

**Affiliations:** 1 Department of Physical Education, Woosuk University, Jeonju, Republic of Korea; 2 Faculty Development Center, Gansu University of Chinese Medicine, Lanzhou, China

**Keywords:** health promotion, physical activity, self-efficacy, social support, university physical education, university students

## Abstract

**Background:**

Whether university physical education courses can support continued participation during the course and initial efforts to remain active beyond it depends on how interpersonal support and organizational opportunities work together. This study examined multilevel support, participation motivation, and psychosocial well-being in university volleyball courses.

**Methods:**

Semi-structured interviews were conducted with 12 students, four instructors, and two administrators from two comprehensive universities in South Korea. The data were analyzed using reflexive thematic analysis.

**Findings:**

Three themes were identified: (1) instructors strengthened students’ confidence to participate by reducing bodily and evaluative threats; (2) peer companionship and shared responsibility for collaboration gave participation relational and value-based significance, although competence comparisons could also lead to withdrawal; and (3) participants linked continuation into extracurricular physical activity to assessment fairness, facility accessibility, campus sport norms, and available post-course pathways.

**Conclusion:**

Participants described continued participation during the course and initial efforts to remain active afterward as more feasible when they felt able and willing to continue and had practical opportunities to do so. University physical education may therefore benefit from linking instructional support, peer interaction, and organizational resources with pathways for activity beyond the course.

## 1 Introduction

Physical inactivity remains a major preventable health risk worldwide. In 2022, approximately 31.3% of adults globally did not meet recommended physical activity levels, indicating that the promotion of regular physical activity cannot rely solely on individual willingness but also requires organized support capable of consistently reaching young adults (1). The university years represent a critical period during which lifestyles are re-established. However, academic pressure, time conflicts, the declining priority assigned to exercise, and disruptions to previous activity habits make it difficult for many students to maintain sufficient physical activity (2, 3). Universities are important public health settings for multilevel physical activity promotion because they provide courses, facilities, and peer networks and can influence health behaviors through institutional arrangements (3, 4). Physical education courses provide students with relatively stable opportunities for physical activity, but their public health value should not be assessed solely in terms of attendance, skill performance, or participation during the semester. Attention should also be given to whether these courses help students develop patterns of physical activity that they are willing to continue, able to continue, and have the conditions necessary to continue. Previous research has shown that high-quality university physical education can improve students’ attitudes toward physical activity and their sport skills (5), while physical activity interventions may also enhance university students’ mental health (6). Nevertheless, completing a physical education course does not necessarily lead to the development of sustained health behavior. Existing research has identified time, resources, social influences, goal prioritization, and competence beliefs as important factors (2, 3). However, there remains limited process-based understanding of how these factors become interconnected through everyday course interactions and why course-based support extends into extracurricular physical activity for some students but ends with the semester for others.

Social support is an important condition linking course experiences to sustained physical activity. A meta-analysis of university students found a consistent positive association between social support and physical activity levels, with support from friends and peers being particularly important (7). Related studies have also suggested that social support may be associated with exercise behavior and positive psychological states through psychological resources such as exercise self-efficacy and self-control (8, 9). However, most existing evidence is derived from cross-sectional surveys or general physical activity contexts and typically examines instructors, peers, and university conditions separately, with limited attention to how these forms of support interact synergistically or conflict within a specific team sport. Team sports combine physical activity, publicly visible skill performance, and interdependence among participants. They may therefore foster belonging, confidence, and psychosocial well-being, while also intensifying psychological pressure through competence comparisons, public mistakes, and unequal access to participation resources (10). Because volleyball skill learning is highly visible and the completion of individual tasks depends heavily on peer coordination, university volleyball provides an analytically informative context for examining these public health-related processes. Against this background, the present study was conducted in elective volleyball courses at two comprehensive universities in South Korea. Drawing on the perspectives of students, teachers, and administrators, it addressed the following research questions:

*RQ1:* How do students experience and understand teacher, peer, organizational, and environmental support in university volleyball courses?

*RQ2:* How is such support related to students’ perceived sport competence, participation motivation, continued engagement in physical activity, and perceived psychosocial well-being?

*RQ3:* Which course arrangements, resource conditions, and aspects of campus culture facilitate or hinder the extension of course participation into physical activity beyond the classroom?

This study aimed to clarify the relationships among interpersonal support, psychological experiences, and organizational opportunities in university physical education and to provide context-sensitive evidence for health-promoting university physical education courses and pathways supporting continued participation after course completion.

## 2 Literature review

### 2.1 Continued physical activity participation and perceived psychosocial well-being

Viewing university physical education courses as settings that connect organized opportunities for physical activity with everyday health behavior, their public health significance lies not only in whether students complete the course but also in whether course experiences support continued participation when students encounter skill-related difficulties, physical discomfort, and competing demands on their time. Here, continued physical activity participation refers to participants’ accounts of maintaining attendance and practice, re-engaging after setbacks, seeking additional practice opportunities, and making initial efforts to remain active beyond the course. It does not indicate that a long-term activity pattern was established. University students’ physical activity is jointly influenced by multiple factors, including competence beliefs, time arrangements, social influences, facility conditions, and behavioral priorities (2, 3, 11). Sustained participation should therefore be understood as a process arising from the interaction between individual action and supportive conditions, rather than as an outcome determined solely by willpower.

The public health value of physical activity also includes the positive psychological and social functioning that participants perceive through participation. Team sports may enhance psychosocial well-being through emotion regulation, experiences of competence, social connection, and group belonging (6, 10). “Perceived psychosocial well-being” refers primarily to participants’ subjective experiences of stress relief, a sense of competence and control, positive relationships, social acceptance, and personal growth. It does not constitute a clinical mental health diagnosis or represent the measurement of all dimensions included in a comprehensive well-being scale. Sustained participation and psychosocial well-being may be mutually reinforcing: positive experiences may increase the appeal of continued participation, whereas stable participation may create further opportunities to develop competence and social connections. However, this study focuses on how participants understand this relationship and does not presuppose a unidirectional or inevitable causal association between the two.

### 2.2 Social support and the multilevel ecology of physical activity

Social support may not only directly provide belonging, recognition, and practical assistance but also mitigate negative experiences when individuals encounter stress or failure (12). In university volleyball courses, support does not consist of a single behavior; rather, it is constituted by multiple sources and functions. Instructors can provide technical information, task structure, evaluative feedback, and emotional reassurance; peers can provide companionship, collaboration, modeling, encouragement, and shared responsibility; and university organizations determine whether students have realistic opportunities for sustained participation through course scheduling, assessment rules, facility access, and pathways to post-course activities.

The social ecological perspective in public health emphasizes that health behavior is simultaneously embedded within individual, interpersonal, organizational, and broader social environments, and that changing any single level is often insufficient to sustain behavior (13). Research on physical activity among university students similarly indicates that social relationships, facility resources, time constraints, identity, and campus norms are not independent influences. Instead, they jointly shape whether students regard physical activity as a feasible, worthwhile, and socially recognized choice (2, 3, 14). Accordingly, this study distinguishes four interrelated levels of support: instructor support, peer support, organizational support, and environmental support, the latter comprising campus sport norms, a climate of physical safety, and recognition of the value of physical activity. This distinction does not imply that the four forms of support can simply be added together. Whether students interpret a particular behavior as supportive also depends on whether the messages conveyed across these levels are consistent. Instructors may encourage students to value progress, but if course assessment rewards only one-time technical performance, the credibility of that encouragement may be weakened. Similarly, peers may be willing to practice together, but when facilities and time are unavailable, emotional support may not translate into actual participation. On this basis, the study uses “coherence of the support ecosystem” as a sensitizing concept to examine whether interpersonal support is reinforced by organizational arrangements and the campus environment. It is not treated as a predetermined conclusion but as an interpretive proposition to be continually examined throughout the analysis.

### 2.3 Self-efficacy: from supportive conditions to the capacity for action

Social cognitive theory defines self-efficacy as an individual’s judgment of their ability to organize and execute a specific course of action (15). Unlike general self-confidence, exercise self-efficacy is explicitly task- and context-specific. Students may recognize the health value of physical activity yet still avoid practice because they doubt their ability to receive the ball, execute a serve, or participate in a match. Self-efficacy influences the tasks individuals choose, the effort they invest, and whether they continue trying after failure.

Self-efficacy judgments are formed primarily through mastery experiences, vicarious experiences, verbal persuasion, and interpretations of physiological and emotional states (15). In physical education, progressive tasks, clear demonstrations, and feedback matched to students’ current ability can help transform complex skills into achievable steps. Comparable peer demonstrations may further enable students to recognize that ability is not fixed. The effectiveness of feedback depends not only on its frequency but also on its content, timing, and how students interpret it (16). Previous research suggests that university and peer support may be associated with physical activity behavior through self-efficacy (8). However, self-efficacy is not an automatic outcome of instructor or peer support. The same corrective feedback may be interpreted as evidence that an instructor believes a student can still improve, or it may be experienced as a public confirmation of insufficient ability. Accordingly, this study conceptualizes the self-efficacy pathway as a process through which students interpret support, bodily experiences, and changes in skill, rather than as a mediating variable mechanically activated by external support.

### 2.4 Motivational internalization: from course requirements to personal health value

Self-determination theory further explains why individuals may be willing to continue a behavior after external demands have diminished. The theory proposes that satisfaction of the three basic psychological needs for autonomy, competence, and relatedness facilitates more autonomous behavioral regulation and positive psychological functioning (17). During the initial stages, participation in university physical education courses may be driven by external requirements such as course credits, assessment, or scheduling. However, extrinsic motivation is not fixed. As students gradually understand the personal value of physical activity, experience improvements in competence, and establish stable connections with others, course requirements may be internalized as “this is meaningful for my health” or “this is a lifestyle I want to maintain.” Motivational internalization is a continuum that varies in degree. Students may participate because they fear losing marks or continue practicing because they feel pressured not to let their teammates down. More autonomous forms of motivation are reflected in recognizing the value of physical activity, enjoying the activity itself, or integrating physical activity into one’s personal identity and way of life (18). Team responsibility therefore does not inherently represent autonomous motivation. It may promote value endorsement through reciprocity, belonging, and shared goals, but it may also create new forms of controlling pressure through guilt, comparison, or fear of negative evaluation. Research among university students has identified an association between perceived social support and autonomous exercise behavior (19), while more autonomous motivation is generally associated with physical activity persistence, enjoyment, and engagement (20). However, the specific effects of instructor, peer, and family support may vary according to the form of support and the context in which it is provided (21). This suggests that what matters more than the source of support itself is whether the support enables students to experience choice, competence, and genuine connection with others.

Self-efficacy and motivational internalization are conceptually distinct: the former addresses the question “Can I do this?”, whereas the latter addresses “Why am I willing to continue doing this?” The two may nevertheless reinforce one another. Successful skill performance can strengthen both competence judgments and experiences of competence, while more autonomous participation motivation may encourage students to practice proactively, thereby creating new mastery experiences. Accordingly, this study does not treat these two pathways as unrelated mechanisms or assume that they unfold in a fixed sequence. Instead, it examines how they intersect, reinforce one another, or become disconnected within specific course experiences.

### 2.5 The volleyball context and an integrative sensitizing framework

Volleyball combines publicly visible physical performance with a high degree of team interdependence. Movement errors, bodily coordination, and differences in skill are often directly visible to instructors and peers, while an individual’s ability to complete a task depends on teammates’ coordination in passing and receiving the ball. Physical pain, fear of the ball, fatigue, and public comparison are therefore not merely skill-related concerns; they also shape students’ judgments of safety, competence, and belonging. Psychological safety in sport settings is associated with coach or instructor behavior, relationship quality, and whether individuals feel able to acknowledge difficulties (22). In volleyball courses, it also concerns whether students can make repeated attempts under conditions in which physical risks are manageable and mistakes are not met with humiliation.

Based on the preceding analysis, this study develops an integrative sensitizing framework linking multilevel social support to continued physical activity participation and perceived psychosocial well-being (Figure 1). Instructor support and peer support constitute the interpersonal resources most directly encountered by students and may operate through two interwoven psychological pathways: (1) support may strengthen sport-specific self-efficacy through task structure, mastery experiences, and competence affirmation, thereby establishing the action foundation for “being able to continue”; and (2) support may facilitate motivational internalization through satisfaction of the needs for autonomy, competence, and relatedness, thereby establishing the value foundation for “being willing to continue.” Organizational arrangements and the campus environment are not treated as peripheral background conditions. Instead, they constitute an opportunity structure that shapes whether support is accessible, credible, and capable of extending into extracurricular physical activity. New experiences generated through sustained participation may, in turn, reshape students’ self-efficacy judgments, endorsement of the value of physical activity, and ways of seeking support.

**Figure 1:**
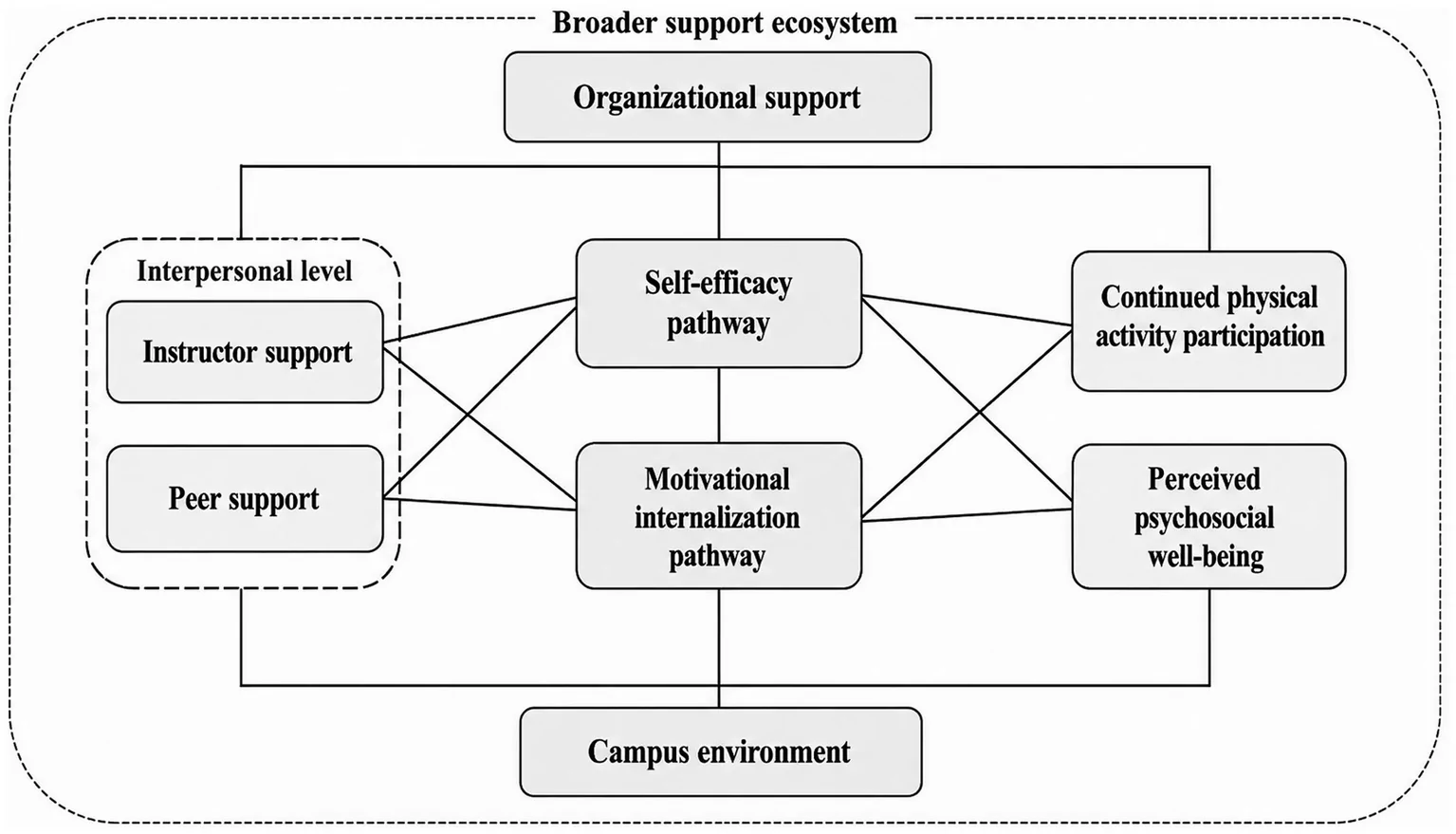
Integrative sensitizing framework for multilevel support and continued participation.

## 3 Methods

### 3.1 Research design

This study employed an interpretivist qualitative descriptive design to understand, from the perspectives of students, teachers, and administrators, how social support in university volleyball courses was perceived and interpreted and how these experiences were related to participants’ accounts of continued physical activity and psychosocial well-being. Qualitative description is well suited to presenting participants’ experiences in language that remains close to their accounts in natural settings and to examining the specific conditions and practical processes through which health behaviors are initiated, maintained, or discontinued (23). Within this interpretivist qualitative descriptive approach, reflexive thematic analysis was used to develop contextually situated interpretations while remaining close to participants’ accounts (24, 25). The analysis followed an abductive logic, with self-efficacy, motivational internalization, and multilevel support providing theoretical sensitivity while remaining open to patterns developed from the data.

### 3.2 Research setting and participants

The study was conducted in elective volleyball courses at two four-year comprehensive universities in South Korea, referred to as KU and SU. The courses were offered to students who were not majoring in physical education and primarily covered forearm passing, setting, serving, spiking, and modified games. This setting was selected because volleyball combines publicly visible bodily performance, skill learning, and team interdependence, thereby providing a context in which interpersonal interactions and organizational conditions could be examined as part of students’ physical activity experiences. Students were recruited through in-class announcements and printed notices at the two universities. Because recruitment was open, the number initially reached could not be determined; 26 students contacted the research team to express interest. Eligible students were adult non-physical-education majors enrolled in the volleyball courses with no or limited previous volleyball experience. Among eligible volunteers, 12 were purposively selected to reflect variation in gender, year of study, academic discipline, and previous volleyball experience. Six instructors and four administrators were approached separately by email; five instructors and three administrators initially agreed to participate. Instructors were involved in elective volleyball teaching, whereas administrators held responsibilities related to physical education or student affairs. One instructor later withdrew because of a scheduling conflict, and one administrator was on leave during data collection, leaving a final sample of 12 students, four instructors, and two administrators. Student accounts formed the primary perspective, while instructors and administrators provided complementary views of the same course support environment.

The study was approved by the Institutional Review Board of Woosuk University. All participants were adults and received written information about the study before the interviews. They provided written informed consent and consent for audio recording. Participation was entirely voluntary, and participants could withdraw at any stage without affecting their course grades, teaching evaluations, employment relationships, or access to university services. To protect participant identities, names, institutional affiliations, and other identifying information were removed from the transcripts. Students, teachers, and administrators were identified using the prefixes S, T, and M, respectively. Only background information relevant to the research questions was reported, and combinations of age, academic discipline, position, and experience that might permit participant identification were avoided. The overall composition of the participant sample is presented in Table 1, and de-identified individual characteristics are provided in Supplementary Table S1.

**Table 1:** Participant characteristics.

Participant group	Number	Gender distribution	Age range	Experience or professional background
Students	12	6 women, 6 men	19–23 years	Four students from each of Years 1, 2, and 3; eight with no previous volleyball experience and four with limited experience
Teachers	4	2 women, 2 men	28–40 years	Backgrounds in volleyball, physical education, sports training, leisure sports, or sport psychology; 3–12 years of teaching experience
Administrators	2	1 woman, 1 man	31–34 years	Responsible for physical education courses, facility coordination, student affairs, or support for sports clubs; 4–6 years of professional experience
Total	18	9 women, 9 men	19–40 years	Three levels of experience represented: students, teaching staff, and organizational administrators

### 3.3 Data collection

Data were collected through individual semi-structured interviews. The interview guides were developed around RQ1–RQ3 and used open-ended questions and follow-up probes concerning social support, perceived sport competence, participation motivation, continued physical activity, and the organizational environment. Questions focused on how participants experienced support and what they believed helped or hindered participation during and beyond the course (26). (1) Student interviews addressed initial participation, skill-related setbacks, teacher feedback, peer collaboration, competence judgments, participation motivation, course assessment, facility conditions, psychosocial experiences, and plans for physical activity after course completion. (2) Teacher interviews focused primarily on instructional support, feedback practices, the design of peer interactions, assessment arrangements, and pathways supporting continued participation after the course. (3) Administrator interviews examined course policies, resource allocation, facility use, the campus sport environment, and organizational support. The complete interview guides are provided in Supplementary Table S2.

The interviews were conducted in Korean by the researchers in private spaces independent of course instruction and grading, thereby reducing the potential influence of the instructional setting on participants’ accounts. Student interviews lasted approximately 45 min on average, whereas interviews with teachers and administrators lasted approximately 55 min. With participants’ consent, all interviews were audio-recorded and transcribed verbatim in Korean. The Korean transcripts were translated into Chinese working texts by bilingual researchers, while the original Korean transcripts remained available throughout the analysis. Key concepts and quotations selected for reporting were checked against the Korean originals to minimize interpretive loss across languages. The complete dataset comprised approximately 185,000 Chinese characters. The contribution of each participant group to the analysis is summarized in Table 2.

**Table 2:** Composition of the interview data.

Data source	Number and duration	Main content	Contribution to the analysis
Student interviews	12 interviews; approximately 45 min on average	Course participation; physical and emotional experiences; teacher feedback; peer collaboration; competence judgments; participation motivation; and extracurricular physical activity plans	To understand how students perceived support and how it was related to sustained participation and psychosocial well-being
Teacher interviews	4 interviews; approximately 55 min on average	Skill scaffolding; feedback practices; psychological and physical safety; group collaboration; assessment; and instructional constraints	To explain the rationale underlying instructional support and the conditions shaping its implementation
Administrator interviews	2 interviews; approximately 55 min on average	Course scheduling; facility use; assessment administration; sport club resources; and continuity of support	To understand how organizational arrangements and the campus environment expanded or constrained opportunities for participation
Total	18 interviews; approximately 185,000 Chinese characters	Experiences of the same course support ecology across three participant groups	To enable comparisons across roles and develop a multilevel interpretation

### 3.4 Data analysis

NVivo 14 was used to manage the data, and Braun and Clarke’s reflexive thematic analysis (24, 25) guided the analysis. The first author conducted initial coding, while all three authors contributed to category and theme development. Analysis involved (1) repeated reading of the Korean transcripts and Chinese working texts with reflexive memoing; (2) coding explicit and underlying meanings, with self-efficacy, motivation, and multilevel support used as sensitizing concepts; (3) comparing codes across participant groups and grouping them into categories; (4) developing candidate themes; (5) reviewing themes against the full dataset, including negative and boundary cases; and (6) defining the final three themes. For example, fear of the ball, task adjustment, and public correction informed physical and evaluative threat, while successful performance and recognition of improvement informed perceived control; together, these categories contributed to the first theme, Teacher support and confidence to continue. Further details are provided in Supplementary Table S3.

### 3.5 Research rigor and reflexivity

Analytical quality was strengthened by addressing credibility, dependability, confirmability, and transferability (27). Throughout the study, versions of the interview guides, coding memos, records of theme revisions, and negative cases were retained to create a traceable analytical audit trail. Theme summaries were returned to three students and one teacher, who were invited to identify interpretations that did not reflect their experiences, relevant experiences that had been omitted, or points requiring reconsideration. Their feedback was treated as additional analytical material rather than as final confirmation of the “correctness” of the themes. The research team also maintained ongoing reflexive records concerning how their theoretical positions, sporting experiences, and value judgments about supportive behavior might have shaped the interviews, coding decisions, and interpretations. Consistent with the interpretivist orientation, these influences were treated as part of the situated interpretive process rather than as factors that could be completely removed from the analysis.

## 4 Findings

Reflexive thematic analysis generated three interrelated themes: (1) teachers created a bodily learning space in which students could attempt skills, make mistakes, and re-engage in the activity by adjusting task difficulty and feedback practices; (2) peer companionship, shared practice, and team responsibility changed the emotional meaning of volleyball participation, although ability comparisons could also lead students to withdraw; and (3) participants linked continuation into extracurricular physical activity to assessment practices, facilities, and the campus sport environment. The three themes and their experiential boundaries are presented in Table 3, with representative and contrasting participant accounts further documented in Supplementary Table S4.

**Table 3:** Theme structure, core experiences, and boundary conditions.

Core theme	Subtheme	Core experience	Negative or boundary experience
Theme 1: A bodily learning space for attempting—moving from fear of the ball toward continued participation	1.1 Reducing physical and evaluative threats	Teachers reduced the risks associated with initial participation through soft equipment, shorter distances, task decomposition, and non-humiliating feedback	Public or frequent correction, or feedback focused only on errors, reduced students’ willingness to attempt the activity
	1.2 Perceived control and confidence to act	Successful skill execution and timely recognition of small improvements helped students begin to believe that their movements could be adjusted	Verbal encouragement alone was difficult to sustain when it was not accompanied by tangible experiences of progress
Theme 2: Changing meanings of shared activity—companionship, responsibility, and comparison pressure	2.1 Emotional companionship and everyday encouragement	Shared practice, relaxed interaction, and peer modeling gave repetitive training social and emotional value	When peers were absent or interactions were weak, individual practice was difficult to sustain
	2.2 Collaborative responsibility and participation identity	Group tasks, instructional roles, and team coordination encouraged students to reconsider their reasons for continuing to participate	Responsibility could also take the form of pressure not to let others down and did not always reflect autonomous choice
Theme 3: The confirmation and withdrawal of support—institutional and environmental conditions shape whether support can be realized	2.3 Ability differences and public comparison	Peers with similar ability levels provided relevant and attainable points of reference for improvement	When ability differences were substantial and roles were not adjusted, comparison weakened students’ perceptions of competence
	3.1 Alignment between teacher and peer support	Teachers designed interactive structures, while peers provided immediate feedback, enabling students to receive confirmation from multiple sources	When group formation lacked deliberate design, peer interaction could magnify ability differences
	3.2 Assessment and resource conditions	Process-oriented assessment, accessible facilities, and appropriate scheduling provided conditions through which students could act on classroom encouragement.	One-off skill assessments, restricted facility access, and scheduling conflicts reduced the perceived value of continued investment
	3.3 Campus norms and post-course continuity	Sport clubs, intramural competitions, and regular practice partners provided pathways for continued participation beyond the course	The dissolution of course groups and disappearance of activity opportunities after course completion led participation to decline rapidly

### 4.1 Teacher support and confidence to continue

For most students with no previous volleyball experience, the initial difficulty involved more than an inability to perform the movements. They also described flinching when the ball approached their bodies, feeling anxious about making mistakes in public, and worrying that they might slow the class down. Teacher support initially took the form of reducing the physical and evaluative risks associated with attempting the activity. S01 explained that the teacher first asked the students to practice forearm passing with balloons and focus only on how the ball contacted the flat surface of their wrists. After completing seven or eight consecutive passes, the student “felt that it was not quite so frightening anymore.” T01 similarly described the priority during the introductory stage as first addressing students’ “fear of the ball” before gradually progressing to standard equipment and complete movements.

Students did not experience all forms of corrective feedback as supportive. Feedback that helped sustain participation usually acknowledged what had already improved while also indicating a feasible next step. After S04 repeatedly served the ball into the net, the teacher did not simply label the movement as incorrect. Instead, the teacher adjusted the serving distance and pointed out that the student’s ball toss was already at an appropriate height. For the student, this response meant that the error remained within a range that could be corrected. By contrast, S08 reported that when the teacher loudly corrected every mistake in front of the entire class, he began remaining at the edge of the practice area and participated less actively.

As practice progressed, students’ perceived control over their bodily movements gradually became an important basis for continued participation. S06 described one successful forearm pass as the moment when he “suddenly felt that I had controlled the ball.” T04 observed that when students unexpectedly completed the correct movement after repeated failures, an immediate confirmation such as “that is the feeling” helped them actively seek the same bodily experience during subsequent practice. Some students also began to shift from waiting for teacher evaluations toward monitoring their own performance. S06 stated that he later became able to assess the position of his wrists after completing a pass. These accounts do not indicate that stable competence had already been established. Rather, they show that students had begun to understand failure as part of an adjustable process and had developed the confidence to continue practicing.

### 4.2 Peer support: companionship, responsibility, and comparison

Peer support changed the emotional experience of repetitive practice. When S07 practiced with a roommate outside their residence hall, one would laugh and retrieve the ball whenever the other misplaced a pass. This relaxed interaction meant that practice was no longer experienced solely as a skill-based task. S10 likewise regarded the time spent practicing and talking with a partner as one of the more relaxing periods of the week and felt that academic pressure was temporarily relieved during the activity. For these students, peers were not merely an emotional resource added to exercise; they were a practical condition that enabled the shared activity to take place.

Peer behavior also provided an everyday impetus for continued participation. S02 initially found practice tiring, but because his roommate regularly arrived early, he gradually felt that he “could not justify being lazy.” Within team tasks, this impetus developed into a sense of responsibility. After being selected as a group leader, S03 initially practiced more because she had to organize the end-of-semester competition. Later, through the experience of being able to help her teammates, she shifted from “having to practice” to “wanting to improve.” While serving as a peer assistant, S09 also began to view herself as “someone who could play volleyball” through demonstrating and correcting movements. S11 joined a university volleyball club after the group competition and was among the few students who extended course participation into organized extracurricular activity.

### 4.3 Conditions for continued participation

When teacher and peer support were connected within the same activity, students were more likely to perceive the feedback as credible. During a paired video-recording exercise, S04 first received confirmation from a partner regarding changes in weight distribution and then additional guidance from the teacher on movement technique. The participant therefore felt that support had come “from both sides.” Teachers also deliberately used grouping arrangements and peer guidance to extend the reach of limited individual feedback. T04 noted that class time was insufficient to attend to every student and that grouping students with complementary ability levels allowed technical points to be reinforced repeatedly through peer interaction.

However, interpersonal support could not substitute for organizational conditions. S12 observed that although the teacher verbally emphasized the importance of improvement, the final grade could still be concentrated on a single serving test. When everyday effort was not reflected in the assessment criteria, she began to question whether continued investment was worthwhile. M01 similarly acknowledged that process-oriented “improvement marks” depended mainly on arrangements made by individual teachers rather than on a standardized policy. Facility conditions created a similar tension. At S10’s university, the outdoor volleyball court lacked lighting at night, while the gymnasium gave priority to varsity team training. The teacher’s encouragement to practice after class was therefore not supported by an accessible space in which students could act on it.

The campus sport environment also shaped whether course participation had a pathway for continuation. S11 interpreted an active volleyball club and intramural competitions as signals that the university recognized continued sport participation among students who were not athletes. By contrast, S04 felt that in a peer environment strongly oriented toward grades and internships, frequent participation in sport could be regarded as “neglecting one’s proper responsibilities.” These differences became more evident after the course ended. S10 explained that the group disbanded immediately after the final competition and that individual practice was difficult to continue without a regular group for arranging games. T04 likewise observed that participation could quickly be interrupted when students had only recently developed interest and confidence but lacked an advanced course, a sport club, or stable practice partners through which to continue. Whether the perceived competence and willingness to participate developed during the course extended into physical activity beyond the course was associated not only with whether students “wanted to continue,” but also with the availability of accessible time, space, social groups, and institutional pathways.

### 4.4 The experiential process of multilevel support and continued participation

Taken together, the three themes informed the development of Figure 2. First, teacher support primarily helped students develop the confidence that they could continue by reducing physical threat, organizing progressive experiences of success, and providing actionable feedback. Second, peer support primarily shaped whether students felt willing to continue through companionship, shared responsibility, and identity affirmation. These two forms of support did not map onto entirely separate pathways. Peer feedback could also influence perceptions of competence, while teacher-created spaces for autonomy could similarly shape students’ motivation to participate. Assessment rules, facility accessibility, campus sport norms, and post-course continuity constituted the opportunity conditions under which interpersonal support could either be realized or withdrawn.

**Figure 2:**
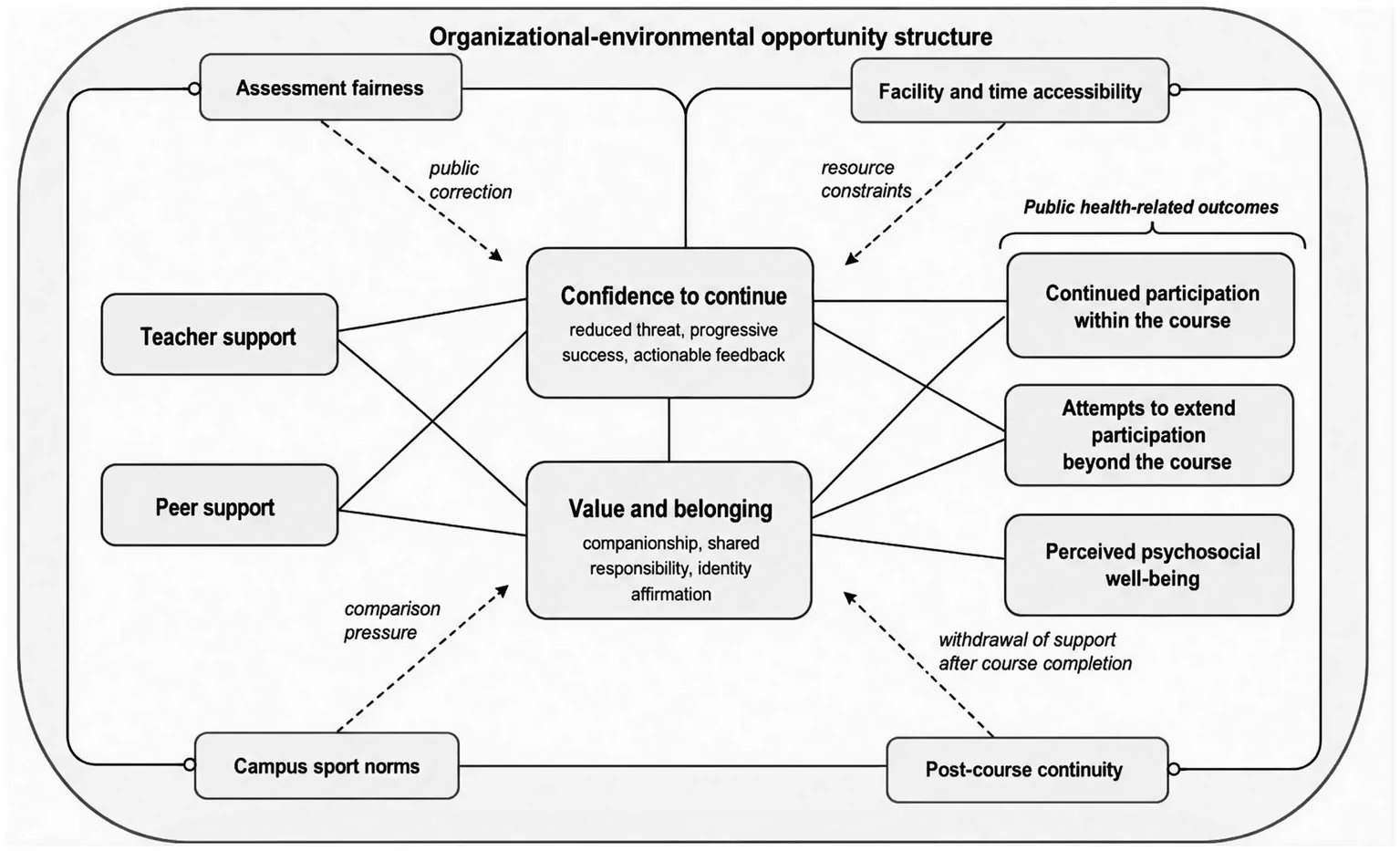
Interpretive model of multilevel support and continued participation.

The model also retained the opposing processes identified in the data. Public correction and ability comparison could reduce students’ willingness to attempt the activity. Outcome-oriented assessment and restricted facility access could undermine the credibility of support. After the course ended, the disappearance of social groups and activity channels could interrupt participation that had only recently begun to take shape. The connections in the figure represent experiential relationships evident in participants’ accounts and should not be interpreted as indicating that this study tested causal relationships among variables.

## 5 Discussion

### 5.1 Bodily safety and confidence to continue

Previous studies have identified competence beliefs, self-regulation, and prior experience as important behavioral conditions for physical activity among university students (2, 3). The present study further indicates that, in beginner volleyball courses, self-efficacy is not an abstract belief in oneself. Rather, it develops when physical threat is controlled, task difficulty can be approached progressively, and small improvements are recognized. According to social cognitive theory, mastery experiences are generally the most influential source of efficacy judgments (15). For students who fear the ball, have limited coordination, or are sensitive to making mistakes in public, however, the opportunity to experience mastery first depends on whether the course allows repeated attempts without excessive risk of injury, embarrassment, or comparison. This interpretation extends research on feedback in physical education, which has primarily focused on feedback formats and skill-learning outcomes (16).

The public health significance of feedback therefore lies not only in improving movement accuracy but also in whether it helps students with lower skill levels remain engaged in physical activity. An exclusive focus on technical standards may increase the frequency of correction while simultaneously intensifying bodily exposure and evaluative pressure. Conversely, general encouragement without task adjustment is unlikely to produce authentic experiences of success capable of sustaining confidence. Supportive physical education should therefore integrate safety, task adaptation, and competence affirmation. What it develops is not short-term optimism, but an actionable judgment that students can adjust their performance and continue participating.

### 5.2 The dual nature of peer support

The positive association between social support and physical activity among university students has received relatively consistent support from systematic reviews and quantitative studies (7, 8, 19). Longitudinal research has also shown that changes in peer support are related to physical activity trajectories as young people transition into university (28). The present study adds an explanation of how this relationship may develop. Peers transformed repetitive practice into a shared everyday activity, giving exercise meanings of enjoyment, companionship, and belonging. Shared tasks also made students feel that their participation affected others, thereby providing additional reasons to attend and remain engaged in practice.

However, not wanting to “let everyone down” should not be treated as equivalent to autonomous motivation. According to self-determination theory, behavior may be sustained through personally endorsed values, but it may also be maintained by guilt, self-imposed pressure, or fear of negative evaluation (17, 18). Peer responsibility therefore had a dual character. When students retained meaningful choice, ability differences were appropriately managed, and individual contributions were recognized, responsibility could develop into perceived value and an emerging sport identity. When ability gaps were substantial, comparisons were public, and students could not leave unsuitable roles, the same responsibility could become a form of controlling pressure. Peer support in team sports should therefore not be treated as a naturally occurring classroom benefit. It requires deliberate design through grouping arrangements, role rotation, and shared goals. The teacher’s role is not limited to providing direct assistance but also includes preventing peer relationships from shifting from mutual support into ability ranking.

### 5.3 Participation quality and psychosocial well-being

Participants’ accounts of stress relief, perceived control, and social connection are consistent with existing evidence that team sports may support psychological and social health (10). Recent qualitative research has similarly shown that whether sport generates positive emotional experiences is closely related to the activity environment, everyday routines, relationships of belonging, and individuals’ interpretations of their sporting experiences (29). The present study indicates that volleyball does not automatically promote psychosocial well-being simply because it involves physical activity. Public humiliation, repeated failure, and negative comparison may produce the opposite experience within the same course. Psychosocial well-being should therefore not be treated as an additional outcome that naturally follows skill acquisition, but as a quality of the participation process itself. When students felt able to control their bodies, believed that mistakes would not damage their relationships, and perceived that their contributions were valued by peers, participation was more likely to be associated with positive emotions and social connection.

Conversely, when a course repeatedly communicated messages of incompetence or non-acceptance, continued attendance could reflect compliance with external requirements rather than a positive experience of participation. This study did not assess clinical mental health outcomes or a comprehensive construct of well-being and therefore cannot establish that the course reduced anxiety or depression or improved overall mental health. The findings nevertheless show that course interactions were important conditions shaping whether students experienced physical activity as restorative, socially connecting, or threatening.

### 5.4 Consistency across the support ecology

The principal contribution of this study to public health research lies in locating teacher and peer support within course arrangements and the wider campus environment. Social ecological theory proposes that health behaviors are jointly shaped by individual, interpersonal, organizational, and environmental levels (13). Research on physical activity has likewise repeatedly identified conditions such as time, facility accessibility, social influences, academic pressure, and university policies (2, 3, 14). The present findings further indicate that these conditions do not merely operate as separate facilitators or barriers; they also shape whether interpersonal support is perceived as credible. When teachers emphasize improvement but rely on a one-off performance assessment, courses require after-class practice without providing accessible facilities, or universities promote health while routinely prioritizing varsity teams over general students in facility use, the health-promoting message loses coherence. “Consistency across the support ecology” therefore offers a more explanatory criterion than the amount of support alone. Public health-oriented physical education should not simply increase teacher encouragement or organize more peer activities. It should also examine whether the values communicated through the course are jointly reinforced by assessment, resources, and opportunities for continued participation. Research on university-based physical activity interventions similarly suggests that participation is more likely to increase when competence, social support, accessible opportunities, and the perceived importance of exercise are addressed together rather than through a single educational message (4). Such consistency does not require every student to receive the same form of activity; it requires the health value promised by the course to be realized through arrangements that are fair, accessible, and responsive to different ability levels.

The withdrawal of support after course completion is particularly important. Previous research has distinguished participation within physical education from physical activity outside class, indicating that completing a physical education course does not mean that students will naturally progress into sustained participation (30). In this study, whether newly developed competence and willingness to participate extended beyond the course was closely related to the availability of regular partners, accessible entry points into sport clubs, facilities, and opportunities for more advanced participation. The public health value of university physical education should therefore not be judged solely by attendance and skill performance during the semester. It should also be assessed by whether the course leaves students with social relationships and organizational pathways that remain available for continued participation.

### 5.5 Theoretical and practical implications

Theoretically, the study integrates three complementary perspectives: the social ecological perspective locates support across interpersonal, organizational, and environmental levels; self-efficacy concerns whether students believe they “can continue”; and motivational internalization concerns why they “want to continue.” The contribution of the support ecosystem concept lies in emphasizing cross-level coherence—whether interpersonal support is reinforced or undermined by organizational arrangements and the campus environment. Thus, “can continue,” “want to continue,” and “have the conditions to continue” represent interrelated conditions rather than a linear causal chain. This interpretation avoids attributing discontinuation of physical activity simply to a lack of individual perseverance and helps explain why students who have positive classroom experiences may nevertheless stop participating after the course ends.

Practically, universities may regard elective physical education courses as health-promotion platforms for the general student population rather than solely as settings for sport skill instruction. (1) Course design should provide beginners with progressively challenging tasks and non-humiliating feedback; (2) peer activities should incorporate complementary ability grouping, role rotation, and shared goals to reduce one-directional comparison; (3) assessment should reflect the participation process, individual improvement, and team collaboration; and (4) facility opening hours, lighting, and reservation systems should be aligned with expectations for after-class practice. Before the course ends, pathways into sport clubs, open recreational activities, or regular practice groups should also be established so that students are not left to locate all necessary resources independently once the formal course is withdrawn. These recommendations represent design directions derived from participants’ experiences and require further implementation or intervention research to assess their feasibility and effects.

### 5.6 Strengths, limitations, and future directions

This study included students, teachers, and administrators, allowing classroom interactions, instructional arrangements, and organizational conditions to be compared within the same analysis. It also retained negative cases, boundary experiences, and accounts of support failure, thereby avoiding the assumption that university physical education courses are necessarily beneficial and showing that the same support arrangements may generate different experiences among students. Several limitations should nevertheless be acknowledged.

1. The data were obtained from two comprehensive universities in South Korea, whose course structures, facility resources, assessment systems, and campus sport norms may differ from those of other regions and types of institutions. The findings should therefore be understood as context-specific interpretations developed within particular settings rather than as statistically generalizable conclusions about university students. Transferability should therefore be considered in relation to comparable institutional, cultural, and physical education contexts. Future research could compare regions, institutional types, and physical education courses to determine which elements of a multilevel support ecology are applicable across contexts and which require adaptation to specific campus conditions.
2. The study relied primarily on one-time retrospective interviews and did not incorporate classroom observations, physical activity monitoring, or longitudinal follow-up after course completion. The temporal sequence among the support, confidence to act, motivational changes, and continued participation described by participants could therefore not be confirmed, nor can the findings establish whether physical activity was maintained over the longer term after the course. Future studies could conduct repeated interviews at the beginning, middle, and end of the semester and after course completion, while incorporating class attendance, facility use, sport club participation, and objective physical activity data to examine temporal relationships between psychological experiences and changes in actual participation.
3. The student participants were primarily beginners or learners with limited experience who remained enrolled in the courses. Students with prolonged absences, those who had withdrawn, and those who actively avoided physical education were not adequately represented. Accounts of teacher support and course assessment may also have been influenced by social desirability. These factors may have resulted in underrepresentation of support failure, exclusion, and withdrawal and may have contributed to a more constructive overall account of course support. Future research should purposively include students who have withdrawn, those with lower physical activity levels, and students from different socioeconomic backgrounds. Anonymous interviews, non-participant observations, or co-design methods could also be used to examine more comprehensively whether course support, assessment systems, and resource accessibility have unequal effects across student groups. Implementation and intervention studies could further test the feasibility of the proposed support arrangements and post-course continuity mechanisms and determine whether they promote sustained physical activity participation in practice.

## 6 Conclusion

Across the interviews, continued participation during the course and initial efforts to remain active afterward were associated with confidence to act, the value attached to participation, and available opportunities. Teacher support contributed to students’ confidence, while peer relationships added companionship and shared responsibility but could also bring comparison and pressure. Beyond the course, participants emphasized the importance of assessment practices, facility access, campus sport norms, and available activity options. University physical education may therefore support health promotion by connecting instructional and peer support with practical opportunities for later participation. Because the study relied on one-time interviews at two South Korean universities, these findings reflect participants’ accounts and do not establish whether physical activity was maintained over the longer term.

## Data Availability

The original contributions presented in the study are included in the article/Supplementary material, further inquiries can be directed to the corresponding author.
