# Supplementary material for: Multilevel support for continued physical activity in university volleyball courses: a qualitative study
Source: Front Public Health. 2026 Sep 25;14:1960528. doi: 10.3389/fpubh.2026.1960528 (PMC13649983; doi:10.3389/fpubh.2026.1960528)
Supplement: Supplementary file 1 [file Table_1.DOCX]

**Additional file 1**

***Table S1. Participant characteristics***

| **ID** | **Gender** | **Age group** | **Year or role** | **Academic or professional background** | **Volleyball experience or years of service** |
| --- | --- | --- | --- | --- | --- |
| S01 | Woman | 19–20 years | Year 1 | Humanities and social sciences | None |
| S02 | Man | 19–20 years | Year 1 | Natural sciences and engineering | None |
| S03 | Woman | 21–23 years | Year 2 | Natural sciences | Limited secondary-school experience |
| S04 | Man | 21–23 years | Year 2 | Humanities and social sciences | None |
| S05 | Woman | 21–23 years | Year 3 | Humanities and social sciences | None |
| S06 | Man | 21–23 years | Year 3 | Engineering | Limited secondary-school experience |
| S07 | Woman | 19–20 years | Year 1 | Natural sciences | None |
| S08 | Man | 19–20 years | Year 1 | Humanities and social sciences | None |
| S09 | Woman | 21–23 years | Year 2 | Humanities and social sciences | Limited club experience |
| S10 | Man | 21–23 years | Year 2 | Natural sciences | None |
| S11 | Man | 21–23 years | Year 3 | Engineering | Limited informal experience |
| S12 | Woman | 21–23 years | Year 3 | Humanities and social sciences | None |
| T01 | Man | 35–40 years | Teacher | Physical education and volleyball | 8 years of teaching experience |
| T02 | Woman | 25–29 years | Teacher | Sports training | 5 years of teaching experience |
| T03 | Man | 35–40 years | Teacher | Leisure sports | 12 years of teaching experience |
| T04 | Woman | 25–29 years | Teacher | Sport psychology | 3 years of teaching experience |
| M01 | Woman | 30–34 years | Academic administrator | Educational administration | 6 years of professional experience |
| M02 | Man | 30–34 years | Student affairs administrator | Social work | 4 years of professional experience |

***Note:*** *To reduce the risk of participant re-identification, exact ages were grouped into age ranges, and academic disciplines and selected aspects of participants’ experience were reported using broad categories.*

**Additional file 2**

***Table S2. Semi-structured interview guide***

| **Participant group** | **Topic** | **Core question** |
| --- | --- | --- |
| Students | Initial participation experience | Please describe how you felt when you first attended the volleyball course. Which people or environmental conditions made you feel safe, nervous, or reluctant to participate? |
| Students | Skill-related setbacks and responses | When you repeatedly failed to serve successfully or could not receive the ball, how did you want the teacher and your peers to respond? What happened in practice? |
| Students | Competence judgments | Please provide examples of teacher or peer behaviors that made you feel either “I can do this” or “I may not be able to do this.” |
| Students | Peer relationships | How did practicing with peers, coordinating during games, or comparing ability levels influence your willingness to continue participating in volleyball? |
| Students | Motivation and value | Why did you initially choose this course? Did your reasons for continuing to participate change as the course progressed? |
| Students | Psychosocial experiences | How did volleyball participation affect your stress, emotions, sense of competence, peer relationships, or sense of belonging on campus? |
| Students | Organizational and environmental conditions | How did assessment methods, course scheduling, facility access, and the campus sport environment influence your participation? |
| Students | Post-course participation | After the course ended, did you continue participating in volleyball or other forms of physical activity? Which conditions supported or hindered this continuation? |
| Students | Negative and boundary experiences | Did you experience any situations that made you want to stop participating or made you feel that support from teachers, peers, or the university had little practical effect? |
| Teachers | Instructional scaffolding | How do you help students with no previous experience, limited coordination, or fear of the ball participate in the course? |
| Teachers | Feedback and safety | How do you usually provide corrective feedback, and how do you respond to differences in students’ reactions to public feedback, physical risk, or failure? |
| Teachers | Peer interaction | How do you organize groups, collaborative practice, and competitions? What forms of assistance or pressure may arise from differences among peers? |
| Teachers | Continued participation | How do you determine whether students are willing to continue participating? What pathways can the course provide for physical activity beyond the classroom? |
| Teachers | Institutional conditions | How do course arrangements, assessment rules, facility resources, and class size support or constrain your teaching? |
| Administrators | Organizational support | What institutional, facility, and staffing support does the university provide for physical education courses and physical activity among the general student population? |
| Administrators | Barriers to participation | What organizational or resource-related difficulties do students commonly encounter when participating in volleyball courses and extracurricular physical activity? |
| Administrators | Assessment and management systems | What principles guide physical education assessment, facility allocation, and safety management? |
| Administrators | Post-course continuity | How are physical education courses connected with sport clubs, intramural competitions, and student health services? |
| Administrators | Areas for improvement | Which organizational or environmental conditions most need to be adjusted to support students’ sustained participation in physical activity? |

**Additional file 3**

***Table S3. Thematic analysis process***

| **Analytical stage** | **Main activities** | **Stage outputs** | **Review focus** |
| --- | --- | --- | --- |
| Data familiarization | Reading the transcripts, checking the translated working texts, and recording preliminary memos | Interview summaries and reflexive records | Whether the researchers understood the backgrounds of participants in different roles and the contexts of specific events |
| Initial coding | Coding explicit statements and underlying meanings relevant to the research questions | Initial code list | Whether experiences extending beyond the conceptual framework were retained |
| Category development | Comparing related codes and interpretations across participant groups | Candidate categories and relational memos | Whether the categories were conceptually distinguishable |
| Candidate theme development | Organizing categories into patterns of meaning capable of addressing the research questions | Preliminary thematic map | Whether the themes extended beyond a simple restatement of the interview questions |
| Theme review | Returning to the complete dataset and comparing positive, negative, and boundary cases | Revised thematic structure | Whether contradictory or inconsistent experiences had been overlooked |
| Theme definition | Clarifying the central argument, scope, and relationships of each theme | Theme definition table | Whether the themes were consistent with the research questions and supporting evidence |
| Analytical writing | Integrating accounts from different participant groups and selecting representative quotations | Results narrative and quotation matrix | Whether participants’ accounts were clearly distinguished from the researchers’ interpretations |

**Additional file 4**

***Table S4. Themes, participants, and representative quotations***

| **Core theme** | **Subtheme** | **Participant** | **Representative quotation** |
| --- | --- | --- | --- |
| Teacher support and confidence to continue | Reducing physical threat | S01 | “In the first class, the teacher had us practice forearm passing with balloons … When I actually completed seven or eight consecutive passes, I felt that it was not so frightening anymore.” |
|  |  | T01 | “The greatest barrier for beginners is fear of the ball … We start with soft balls and then break the movement down. Once students realize that they can complete the first step, they become willing to try the next.” |
|  | Non-humiliating correction | S04 | “The teacher did not say, ‘That is wrong.’ Instead, she asked me to step back two paces … She said that the height of my toss had actually been good. That made me feel that making mistakes was acceptable.” |
|  | Negative experience of public correction | S08 | “Whenever I made a mistake, the teacher called it out in front of everyone … The whole class turned to look at me. After that, I only dared to stay in the corner for the rest of the class.” |
|  | Perceived bodily control | S06 | “When I passed the ball and watched it land steadily on the other side of the court … I suddenly felt that I had controlled the ball.” |
|  | Timely confirmation | T04 | “The eleventh attempt succeeds. If I nod at that moment or say, ‘That is the feeling,’ the student will remember the success that has just occurred.” |
|  | Recognition of small improvements | S03 | “The teacher quietly said, ‘Your movement just now was much faster than last week’ … I practiced with particular enthusiasm for the rest of the class.” |
| Peer support: companionship, responsibility, and comparison | Emotional companionship | S07 | “We often practiced forearm passing outside our residence hall. When one of us misplaced a pass, the other would laugh and run over to retrieve the ball.” |
|  | Stress relief | S10 | “When I practiced passing with my partner and we talked about different things, it felt as though the stress of writing my paper was being passed away with the ball.” |
|  | Behavioral encouragement | S02 | “My roommate always arrived early to reserve a place on the court. Gradually, I felt too embarrassed to slack off.” |
|  | Group engagement | T03 | “When even one or two members of a group practice seriously, the level of engagement across the whole group increases.” |
|  | Collaborative responsibility | S03 | “At first, I practiced only because I had been selected as the group leader and had to take responsibility. Later … it changed from ‘I have to practice’ to ‘I want to improve.’” |
|  | Instructional role and self-understanding | S09 | “I was not only helping others; I was also redefining myself—as though I had really become ‘someone who could play volleyball.’” |
|  | Extension beyond the course | S11 | “When everyone exchanged high-fives after the competition … that moment made me decide that I wanted to continue playing the following semester, so I later joined the university volleyball club.” |
|  | Comparison pressure | S08 | “My partner had played volleyball since childhood … She received the ball effortlessly while I was flustered. I felt extremely discouraged and even considered changing groups.” |
| Conditions for continued participation | Alignment between teacher and peer support | S04 | “After first hearing encouragement from my partner and then receiving guidance from the teacher, I felt supported by both of them.” |
|  | Instructional organization of peer support | T04 | “I pay attention to group composition so that students who learn more quickly can support their peers … Technical points can then be reinforced repeatedly through peer interaction.” |
|  | Inconsistency in assessment | S12 | “The teacher encouraged us by saying that ‘improvement is more important than the grade,’ but if the final grade depends only on a single serving test, what difference does all the effort during the semester make?” |
|  | Variation in assessment practices | M01 | “Some teachers independently include ‘improvement marks,’ but this is not a standardized requirement.” |
|  | Facility constraints | S10 | “The outdoor volleyball court has no lighting at night … The teacher encouraged us to practice after class, but there was nowhere to practice. The encouragement therefore felt empty.” |
|  | Resource allocation | M02 | “Sport facilities give priority to varsity team training, so the time available to students enrolled in elective courses is indeed limited.” |
|  | Supportive campus sport norms | S11 | “The university has an active volleyball club and regularly organizes intramural competitions. This made me feel that playing volleyball was recognized and encouraged on campus.” |
|  | Questioning the value of sport | S04 | “Everyone around me is competing for higher grades and better internships. If you play sport every day, it can look as though you are not taking your proper responsibilities seriously.” |
|  | Withdrawal of support after the course | S10 | “After the final competition, everyone said, ‘See you next semester,’ and then the group dispersed. Without a regular group for arranging games, it is difficult to continue alone.” |
|  | Lack of a continuation platform | T04 | “By the end of the course, students may have only just developed some confidence and interest. Without a subsequent platform through which to continue, their participation can quickly stop.” |
